# Adverse childhood experiences and adolescent cannabis use trajectories: findings from a longitudinal UK birth cohort

**DOI:** 10.1016/S2468-2667(23)00095-6

**Published:** 2023-05-25

**Authors:** Lindsey A Hines, Hannah J Jones, Matthew Hickman, Michael Lynskey, Laura D Howe, Stan Zammit, Jon Heron

**Affiliations:** a Department of Psychology, University of Bath, Bath, UK; b Bristol Medical School, University of Bristol, Bristol, UK; c Centre for Academic Mental Health, Bristol Medical School, University of Bristol, Bristol, UK; d Population Health Sciences, Bristol Medical School, University of Bristol, Bristol, UK; e UK Medical Research Council (MRC) Integrative Epidemiology Unit at the University of Bristol, Bristol, UK; f UK NIHR Biomedical Research Centre, University Hospitals Bristol NHS Foundation Trust, University of Bristol, Bristol, UK; g Drug Science, London, UK; h Division of Psychological Medicine and Clinical Neurosciences, MRC Centre for Neuropsychiatric Genetics and Genomics, Cardiff University School of Medicine, Cardiff, UK

## Abstract

**Background:**

Adverse childhood experiences (ACEs) are classically defined as physical abuse, sexual abuse, emotional abuse, emotional neglect, bullying, parental substance use or abuse, violence between parents, parental mental health problems or suicide, parental separation, or a parent convicted of criminal offence. Exposure to ACEs can be associated with cannabis use, but no comparisons across all adversities have been made while also considering timing and frequency of cannabis use. We aimed to explore the association between ACEs and cannabis use timing and frequency in adolescence, considering the cumulative number of ACEs and individual ACEs.

**Methods:**

We used data from the Avon Longitudinal Study of Parents and Children, a longitudinal UK birth cohort study. Longitudinal latent classes of cannabis use frequency were derived from self-reported data at multiple timepoints in participants aged 13–24 years. ACEs between ages 0 years and 12 years were derived from prospective and retrospective reports at multiple timepoints by parents and the participant. Multinomial regression was used to analyse the effect of both cumulative exposure to all ACEs and the ten individual ACEs on cannabis use outcomes.

**Findings:**

5212 participants (3132 [60·0%] were female and 2080 [40·0%] were male; 5044 [96·0%] were White and 168 [4·0%] were Black, Asian, or minority ethnic) were included in this study. After adjustment for polygenic risk and environmental risk factors, participants who had 4 or more ACEs at age 0–12 years were at increased risk of early persisting regular cannabis use (relative risk ratio [RRR] 3·15 [95% CI 1·81–5·50]), later onset regular use (1·99 [1·14–3·74]), and early persisting occasional use (2·55 [1·74–3·73]) compared with low or no cannabis use. After adjustment, early persisting regular use was associated with parental substance use or abuse (RRR 3·90 [95% CI 2·10–7·24]), parental mental health problems (2·02 [1·26–3·24]), physical abuse (2·27 [1·31–3·98]), emotional abuse (2·44 [1·49–3·99]), and parental separation (1·88 [1·08–3·27]) compared with low or no cannabis use.

**Interpretation:**

Risks for problematic adolescent cannabis use are highest for individuals reporting 4 or more ACEs, and were particularly raised for those with parental substance use or abuse. Public health measures to address ACEs might reduce adolescent cannabis use.

**Funding:**

The Wellcome Trust, UK Medical Research Council, Alcohol Research UK.

## Introduction

Adolescent cannabis use can be associated with psychiatric disorders,^1^, ^2^ and the prevalence of cannabis use disorder has been increasing in countries such as the USA.^3^ Consequently, cannabis use in adolescents is an important public health issue. As cannabis policy evolves and the availability of cannabis and acceptability of use is likely to increase, it is important in terms of interventions to understand who is at risk of developing problematic patterns of cannabis use.

One area of focus is adverse childhood experiences (ACEs),^4^ defined as physical abuse, sexual abuse, emotional abuse, emotional neglect, bullying, parental substance use or abuse**,** violence between parents, parental mental health problems or suicide, parental separation, or a parent convicted of criminal offence. ACEs cluster,^5^ and outcomes are worse for those who have more ACEs.^6^ Consequently, 4 or more ACEs are seen as an indicator of increased risk of negative health outcomes.^6^ A systematic search identified that some studies have explored a selection of the aforementioned adversities in relation to cannabis use, and many have considered the effect of multiple ACEs (see summary table of literature, appendix pp 2–4). Of three studies^7^, ^8^, ^9^ that allowed consideration of the effects of individual ACEs, findings were mixed. There was no strong correlation between child abuse or neglect and cannabis use in adolescence,^7^ whereas the findings from two other studies reported that sexual abuse and witnessing violence among those aged 11–17 years increased the likelihood of cannabis use in adolescence.^8^, ^9^ However studies^8^, ^9^ disagree on the effects of living with a parent with an alcohol problem. A 2021 review of the relationship between sexual abuse in childhood and cannabis use in adolescence reported adjusted odds ratios of 0·53–2·18 in 11 studies, partly owing to differences in the conceptualisation of sexual abuse and the information sources used.^10^ Such differences might also arise due to the age groups studied (appendix pp 2–4); many studies of ACEs and substance use or abuse focus on secondary school and early adolescence, which might not fully capture the time period required for substance use or abuse to establish.

**Research in context**

**Evidence before this study**

We searched abstracts of journal articles on MEDLINE, PsycINFO, and Embase from database inception to Aug 9, 2022, with no restriction on language, using the terms (“cannabis” or “marijuana”) and (“adolescen*”) and (“ACEs” or “adverse childhood experiences”); for more information see the appendix (pp 2–4). Epidemiological studies were included if they used one or more of the 10 classic adverse childhood experiences (ACEs) as the exposure, included adolescent cannabis use as an outcome, and used a general population human sample. The search identified 15 papers, of which two were excluded. Meta-analysis was not possible due to heterogeneity of exposures, but there is a consistent trend towards increased likelihood of adolescent cannabis use after exposure to multiple ACEs. Findings relating to individual ACEs are not consistent, which is probably due to differences in how and when ACEs are measured, and how cannabis use is conceptualised. Many studies have considered the effect of multiple ACEs (appendix pp 2–4), but no previous studies have provided a comparison of the effects of all ten individual ACEs alongside a cumulative measure of ACE exposure. Differences in effect estimates in previous studies might have arisen because of short follow-ups; many studies of ACEs and substance use or abuse focus on secondary school (approximately age 13–18 years), which might not fully capture the period of substance use initiation.

**Added value of this study**

The present study explores the association between ACEs and cannabis use in a sample in which ACEs data were collected thoroughly, both prospectively and retrospectively at multiple timepoints. Cannabis use frequency data were collected prospectively as multiple timepoints, allowing for consideration of both timing and frequency of use, which is important because earlier use of cannabis in youth might be associated with adverse effects. To our knowledge, no studies have allowed for comparison of effects between each of the individual ACE exposures, and dose–response effect, and no studies have taken into account the plausibly confounding influence of parental substance use and genetic risk for substance use. The present study highlights which specific ACEs might be promising targets for intervention to reduce cannabis use in adolescence.

**Implications of all the available evidence**

Experiencing multiple adversities raises risks for cannabis use, and public health efforts to reduce ACEs could reduce problematic cannabis use during adolescence. Additionally, the present findings relating to parental substance use or abuse suggest children growing up in such households might be an important group for targeted intervention. There is a need to consider supportive interventions across the lifecycle, and with parents, to reduce cannabis use in adolescence.

Our work builds on the existing literature. The Avon Longitudinal Study of Parents and Children (ALSPAC), a large longitudinal UK population-based cohort, includes repeated prospective and retrospective measures of 10 ACEs, with data collection from birth.

Deriving cannabis use from repeated measures with a data-driven method allows for consideration of timing and frequency of cannabis use. This consideration is overlooked in the majority of studies in this area^10^ but is crucial given that the likelihood of harms associated with cannabis use might be greater when onset of use is earlier^11^ and use is frequent.^12^ Longitudinal data can be used to identify patterns of the frequency of cannabis use and onset in adolescents. Although studies of cannabis trajectories indicate heterogeneity between studies,^12^, ^13^, ^14^ trajectories from adolescence to adulthood commonly identify groups with low cannabis use, chronic regular use, and declining use. Using trajectories avoids making a priori assumptions about patterns of use in a population.

Previous studies have not been able to control for the potentially confounding effects of genetics, and parental mental health problems and substance use or abuse. There is genetic liability to substance use or abuse,^15^ and research has identified that the genetic propensity to initiate cannabis use is associated with different patterns of adolescent cannabis use;^13^ given that parental substance use or abuse is not only an adversity, but is also linked to perpetuation of other ACEs,^16^ it is plausible that genetics for substance use or abuse might confound the association. Similarly, parental mental health problems and substance use or abuse are factors associated with cannabis use in children.^17^ Cannabis use in adolescence might be affected indirectly by a parent's history of mental health problems and substance use or abuse; parental mental health before pregnancy can influence their child's mood;^18^ and genes,^19^ family environment,^19^ and parental attitudes to drug use can contribute to the transmission of substance use disorders.^20^ By adjusting for the effect of parental substance use or abuse and mental health problems before the child's birth (which is not considered an ACE, occurring before the child is born), the extent to which the likelihood of adolescent cannabis use is directly due to childhood exposure to ACEs distinct from these other mechanisms of substance use transmission can be estimated.

Using the UK population-based ALSPAC cohort, the present study aimed to describe trajectories of cannabis use in individuals aged 13–24 years, explore the association between ACEs and cannabis trajectory classes (considering both the cumulative number of ACEs and the individual ACEs), and identify the extent to which these associations are attenuated by genetic and environmental risk factors.

## Methods

### Study design and participants

We used data from ALSPAC, a UK population-based birth cohort. In brief, pregnant women in the South West of England with an estimated date of delivery between April 1, 1991, and Dec 31, 1992, were invited to take part via media campaigns and outreach via antenatal and maternity services. For further details on recruitment, see the appendix (p 5); full details have been published elsewhere.^21^, ^22^, ^23^ Ethics approval for this study (project proposal B2881) was obtained from the ALSPAC Law and Ethics Committee and the Local Research Ethics Committees. Informed consent for the use of data collected via questionnaires and clinics was obtained from participants following the recommendations of the ALSPAC Ethics and Law Committee at the time. Study participation was voluntary, and during data collection information was provided on the intended use of data; consequently, returning a questionnaire or attending a clinic was considered written consent. However, for collection of biological samples from parents or children, the parents completed a written consent form. Consent for the use of biological samples was collected in accordance with the Human Tissue Act (2004). Participants can contact the study team to retrospectively withdraw consent for their data to be used in research at any time.

The present analyses were nested within a longitudinal birth cohort. The sample was limited to participants who provided self-reported data on cannabis use in adolescence (age 13–24 years) and participants were included whether they responded “yes” or “no” to questions on past-year cannabis use. Participants had to have provided a response at one or more (out of six possible) data collection points between ages 13 years and 18 years, and at one or more (out of three possible) data collection points between ages 20 years and 24 years. It was necessary to include those who reported not using cannabis to observe variation in patterns of use over time.

### Procedures

Data on cannabis use were collected at nine timepoints from age 13–24 years, through child-completed questionnaires. Data on ACEs were collected at 68 timepoints between a woman giving birth and her child reaching age 18 years. Data collection methods included 34 child-completed questionnaires, nine focus clinical assessments, and 25 questionnaires about the child completed by the mother or other main caregiver. For further details see the appendix (p 5); full details have been published elsewhere.^21^, ^22^, ^23^

### Exposure

In the present analyses, the period of exposure to ACEs was set as age 0–12 years to ensure this exposure preceded cannabis use measures included in the trajectories. The following ACEs were included: physical abuse, sexual abuse, emotional abuse, emotional neglect, bullying, parental substance use or abuse (including alcohol abuse), interpartner violence between parents, parental mental health problems or suicide, parental separation, and a parent convicted of criminal offence. In ALSPAC, 377 separate variables contribute to these ACE exposures at age 0–12 years, and these variables were taken from both mother and child reports; if reports did not agree, an ACE was assumed present if it was reported at any timepoint by any respondent. Not all ACEs were reported at the same timepoints. For each of the ACEs, binary exposure variables were derived. Given the number of timepoints involved in the data, participants who had responded to more than 50% of the questions for a specific ACE were included in the binary measure. Participants who had responded to less than 50% of the questions were coded as missing (further details of derivation and definitions are in the appendix pp 5–11).^24^ After multiple imputation, composite dummy variables were derived of participants having 0, 1, 2–3, or 4 or more ACEs to explore dose–response effects of ACEs on cannabis use; three separate variables for the number of ACEs reported were derived, with the reference group coded as reporting 0 ACEs and all other levels of ACE exposure. For example, the dummy variable for 4 or more ACEs has a reference group of 0 ACEs, 1 ACE, and 2–3 ACEs.

### Outcomes

The primary outcome was patterns of cannabis use frequency and onset in adolescence. Repeated measures of the frequency of cannabis use were used to derive trajectories of cannabis use. Data were collected through self-report questionnaire at nine timepoints throughout adolescence. Responses were at approximately 13 years, 14 years, 15 years, 16 years, 17 years, 18 years, 20 years, 22 years, and 24 years, and were derived as a repeated three-level ordinal variable with categories ”do not use”, “occasional” (typically less than once per week), and “regular” (typically once a week or more; appendix p 16).

### Covariates

Covariates reported by mothers and their partners before the birth of the participant were maternal or partner substance use during pregnancy, maternal or partner mental health before or during pregnancy, and financial difficulties. Full information on covariates is provided in the appendix (pp 11–12).

Biological sex of participants was reported at birth. Childhood socioeconomic position data were collected through parental report when participants were aged 8 months, 21 months, 33 months, 4 years, and 8 years. Weighted cannabis initiation polygenic scores were constructed in participants for cannabis initiation using results from the cannabis use genome-wide association study meta-analysis^25^ with data from the International Cannabis Consortium and UK Biobank (n=184 765).

### Statistical analysis

The analysis was not registered in advance of the study and the results should be considered exploratory. For transparency in the multiplicity of exposures, the correlation matrix for exposures is reported in the appendix (p 17). The highest correlation was 0·27 (between emotional abuse and physical abuse).

Trajectories of cannabis use were derived using longitudinal latent class analysis in Mplus version 8.3. To establish the optimal number of latent classes, we used: (1) the sample size-adjusted Bayesian information criterion, (2) the bootstrap likelihood ratio test, (3) entropy, and (4) bivariate model fit information.

Associations between ACEs and cannabis trajectory class were estimated as relative risk ratios (RRRs) with 95% CIs using multinomial logistic regression. ACE exposure was entered as three dummy variables for cumulative number of ACEs (with each level of cumulative exposure compared against all other levels), and each individual ACE was included in a separate model. This approach resulted in 13 univariable regression models and 13 multivariable regression models (26 models).

The normative latent class was used as the reference category for the outcome; this emerged as a class of low or no cannabis use. Parameter estimates were obtained using the Modal ML three-step method.^26^ This approach has been shown to produce less biased estimates than traditional three-step classify–analyse methods while avoiding the problem of covariates affecting the measurement model itself. Omnibus Wald tests of pairwise comparisons were applied to assess differences between classes.

To maintain temporality, models were adjusted for covariates only if they preceded both onset of ACEs and cannabis use: mother or partner mental health before or during pregnancy, mother or partner substance use during pregnancy, and financial difficulties during pregnancy. Models were also adjusted for participants’ cannabis polygenic score, socioeconomic position, and biological sex assigned at birth.

We derived the latent classes first in MPlus, and applied full information maximum likelihood estimation for this model to maximise the use of available data on cannabis; consequently, imputation of the outcome data was not required.

Missing data in exposure and covariate measures were addressed through multiple imputation in Stata (version 16.1) using chained equations: a series of univariable regression models that impute each incomplete variable sequentially (mi impute chained), before converting the data back into MPlus format to run the regression analyses. MPlus data were registered as imputed to run the analyses over the 40 imputed data files. Details on the imputation models are in the appendix (p 13).

### Role of the funding source

The funders had no role in the study design, data collection, analysis, data interpretation, writing of the report or decision to submit the manuscript.

## Results

**Figure 1:**
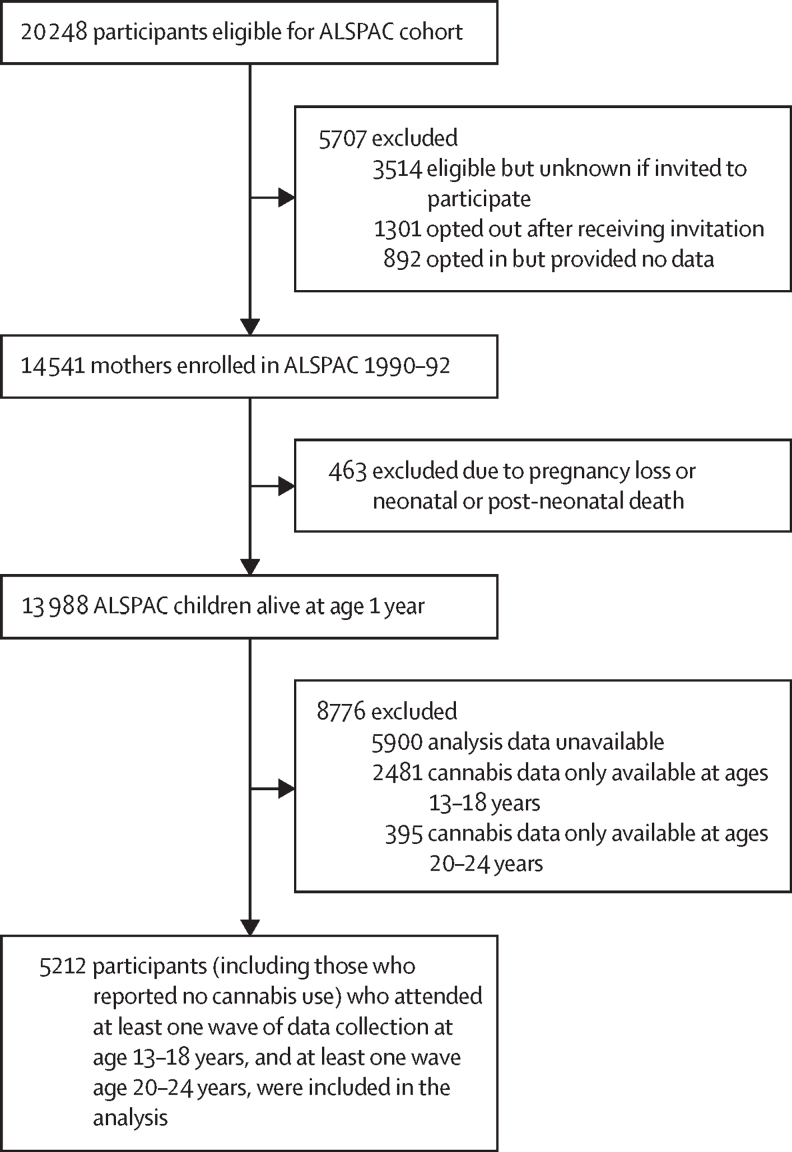
Flow diagram for study sample ALSPAC=Avon Longitudinal Study of Parents and Children.

14 541 mothers who delivered between April 1, 1991, and Dec 31, 1992, were enrolled in ALSPAC, and 13 988 children of these mothers were alive at 1 year. Following exclusion of 8776 participants, 5212 participants (3132 [61·1%] female and 2080 [39·9%] male; 5044 [96·0%] were White and 168 [4·0%] had Black, Asian, or minority ethnicity) who provided data on cannabis use at age 13–18 years and age 20–24 years were included in this study (figure 1). Sample characteristics are shown in table 1. Of the 5212 participants in the sample, after imputation an estimated 3731 (71·6%) had at least 1 ACE between ages 0 years and 12 years: 1532 (29·4%) reported 1 ACE, 1657 (31·8%) reported 2–3 ACEs, and 542 (10·4%) reported 4 or more ACEs. The most prevalent adversity was having at least one parent who had experienced a mental health problem or suicide attempt. The appendix show details on the number of ACEs in participants who reported 4 or more (p 17) and the distribution of individual ACEs within according to participants who had one ACE, 2–3 ACEs, and 4 or more ACEs (p 18).

**Table 1:** Sample characteristics in imputed data for the 5212 participants

		**Proportion of participants (%)**
**Exposures (age 0–12 years)**		
No ACEs		1481* (28·4%)
Any ACEs		3731* (71·6%)
	1 ACE	1532* (29·4%)
	2–3 ACEs	1657* (31·8%)
	4 or more ACEs	542* (10·4%)
Parental substance use or abuse		469* (9·0%)
Parental mental health problems or suicide attempt		2168* (41·6%)
Parent with criminal conviction		370* (7·1%)
Parental separation		1095* (21·0%)
Interpartner violence between parents		980* (18·8%)
Bullying		662* (12·7%)
Physical abuse		714* (13·7%)
Sexual abuse		162* (3·1%)
Emotional abuse		1006* (19·3%)
Emotional neglect		318* (6·1%)
**Covariates**		
Sex		
	Female	3132 (60·1%)
	Male	2080 (39·9%)
Lower socioeconomic position at age 0–12 years		380* (7·3%)
Maternal substance use during pregnancy		33 (0·6%)
Maternal partner substance use during pregnancy		246 (4·7%)
Maternal mental health problems before or during pregnancy		557 (10·7%)
Maternal partner mental health problems before or during pregnancy		309 (5·9%)
Financial difficulties during pregnancy		2811 (53·9%)
**Additional data**		
Ethnicity		
	Black, Asian, or minority ethnicity	168 (4·0%)
	White	5044 (96·0%)

Data are n (%). ACE=adverse childhood experience.

* Estimated from imputed proportions as data were incomplete.

Participants excluded due to missing waves of data collection were similar to the analysis sample, but those with complete data were more likely to be female, White, and in a higher socioeconomic position (appendix pp 19–20). The proportion of missing data on exposures and covariates in the sample ranged from 0% to 53·6%, but the proportions of characteristics were similar in both complete case and imputed datasets (appendix p 21).

**Figure 2:**
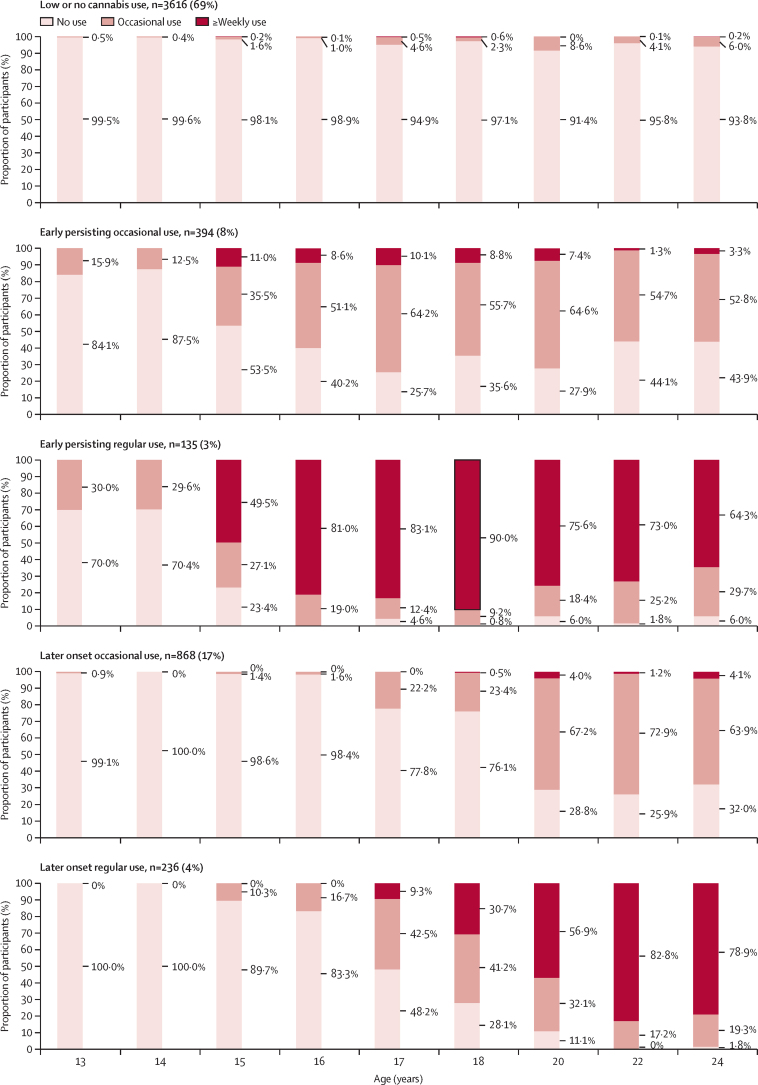
Cannabis trajectory classes showing timing and frequency of use at age 13–24 years (derived from repeated measures)

The prevalence of past-year cannabis use increased between age 13 years and 20 years and began to plateau at age 24 years (table 2; appendix p 22 shows these data in those who attended all nine waves of data collection). In the latent class analysis a five-class solution explained the heterogeneity in the data on cannabis use frequency; this solution replicated across multiple patterns of missing data (appendix p 23). On the basis of measures of Bayesian information criterion, replicated log-likelihood, and Lo-Mendell-Rubin test p values, a six-class solution was considered, but rejected in favour of the five-class solution because the six-class model included one class for which interpretation was questionable. The five-class solution identified trajectories of cannabis use, which were labelled low or no cannabis use (3616 [69%] participants), later onset occasional use (868 [17%] participants), early persisting occasional use (394 [8%] participants), later onset regular use (236 [4%] participants), and early persisting regular use (135 [3%] participants; figure 2).

**Table 2:** Prevalence of past-year cannabis use at each data collection timepoint using all estimated available data

	**Age 13 years (n=5710)**	**Age 14 years (n=5599)**	**Age 15 years (n=4960)**	**Age 16 years (n=4804)**	**Age 17 years (n=3850)**	**Age 18 years (n=3178)**	**Age 20 years (n=3967)**	**Age 22 years (n=3641)**	**Age 24 years (n=3527)**
Do not use	5516 (96·6%)	5460 (97·5%)	4483 (90·4%)	4330 (90·1%)	3113 (80·9%)	2661 (83·7%)	2780 (70·1%)	2711 (74·5%)	2536 (71·9%)
Occasional use	176 (3·1%)	106 (1·9%)	329 (6·6%)	317 (6·6%)	569 (14·8%)	371 (11·7%)	973 (24·5%)	748 (20·5%)	771 (21·9%)
Regular use	18 (0·3%)	33 (0·6%)	148 (3·0%)	157 (3·3%)	168 (4·4%)	146 (4·6%)	214 (5·4%)	182 (5·0%)	220 (6·2%)

Data are n (%).

Unadjusted and adjusted results are presented in table 3. After adjustment for maternal or partner drug use during pregnancy, maternal or partner mental health before or during pregnancy, family financial difficulties before the participant's birth, biological sex of participant reported at birth, childhood socioeconomic position at age 0–12 years, and participants’ cannabis initiation polygenic risk scores, participants who had 4 or more ACEs were at more than three times the risk of early persisting regular use (RRR 3·15 [95% CI 1·81–5·50]; p<0·001), at twice the risk of later onset regular use (1·99 [1·14–3·46]; p=0·016), and at more than twice the risk of early persisting occasional use (2·55 [1·74–3·73]; p<0·001) relative to the low or no cannabis use class. Those who had 2–3 ACEs were at increased risk of later onset regular use (RRR 1·69 [95% CI 1·14–2·53]; p=0·010) and early persisting occasional use (1·52 [1·12–2·06]; p=0·007) relative to the low or no cannabis use class, with weaker evidence indicating increased risk of early persisting regular use (1·46 [0·91–2·35]; p=0·119).

**Table 3:** Multinomial regression associations between exposure to ACEs at age 0–12 years and cannabis use frequency at age 13–24 years

	**Unadjusted RRR of early persisting regular use, n=135 (95% CI); p value**	Adjusted RRR of early persisting regular use, n=135*(95% CI); p value	**Unadjusted RRR of later onset regular use, n=236 (95% CI); p value**	Adjusted RRR of later onset regular use, n=236*(95% CI); p value	**Unadjusted RRR of early persisting occasional use, n=394 (95% CI); p value**	Adjusted RRR of early persisting occasional use, n=394*(95% CI); p value	**Unadjusted RRR of later onset occasional use, n=868 (95% CI); p value**	Adjusted RRR of later onset occasional use, n=868*(95% CI); p value	**Low or no cannabis use, n=3616 (reference class)**
**Cumulative ACE exposures age 0–12 year**s									
One ACE (n=1508);† dummy variable reference: no ACEs, 2–3 ACEs, 4 or more ACEs	0·59 (0·34–1·01); p=0·054	0·69 (0·39–1·19); p=0·181	0·62 (0·39–1·00); p=0·051	0·64 (0·39–1·05); p=0·080	0·74 (0·53–1·03); p=0·074	0·75 (0·53–1·06); p=0·105	0·93 (0·71–1·22); p=0·617	0·95 (0·72–1·25); p=0·710	1·0 (ref)
2–3 ACEs (n=1657);†‡ dummy variable reference: no ACEs, 1 ACE, 4 or more ACEs	1·70 (1·09–2·65); p=0·02	1·46 (0·91–2·35); p=0·119	1·70 (1·15–2·50); p=0·008	1·69 (1·14–2·53); p=0·010	1·60 (1·19–2·14); p=0·002	1·52 (1·12–2·06); p=0·007	1·13 (0·86–1·48); p=0·372	1·13 (0·86–1·50); p=0·385	1·0 (ref)
4 or more ACEs (n=542);†§ dummy variable reference: no ACEs, 1 ACE, 2–3 ACEs	4·31 (2·64–7·04) p≤0·001	3·15 (1·81–5·50) p≤0·001	2·32 (1·43–3·74) p≤0·001	1·99 (1·14–3·46) p=0·016	2·65 (1·84–3·82) p≤0·001	2·55 (1·74–3·73) p≤0·001	1·17 (0·75–1·83) p=0·47	1·16 (0·72–1·86) p=0·542	1·0 (ref)
**ACE exposure**									
Parental substance use or abuse (n=469)§	7·16 (4·38–11·68); p≤0·001	3.90 (2·10–7·24); p≤0·001	2·62 (1·54–4·46); p≤0·001	1·82 (0·97–3·40); p=0·061	3·07 (2·03–4·63); p≤0·001	2·38 (1·51–3·73); p≤0·001	2·02 (1·34–3·05); p≤0·001	1·81 (1·15–2·83); p=0·01	1·0 (ref)
Parental mental health problems or suicide attempt (n=2168)¶	2·39 (1·54–3·70); p≤0·001	2·02 (1·26–3·24); p=0·003	1·29 (0·91–1·85); p=0·15	1·20 (0·81–1·76); p=0·36	1·52 (1·15–2·00); p=0·003	1·50 (1·11–2·03); p=0·008	1·16 (0·91–1·48); p=0·24	1·15 (0·88–1·49); p=0·305	1·0 (ref)
Parent with criminal conviction (n=370)	1·33 (0·57–3·08); p=0·51	1·15 (0·47–2·81); p=0·754	1·31 (0·62–2·77); p=0·48	1·23 (0·55–2·74); p=0·607	1·91 (1·22–3·01); p=0·005	1·90 (1·20–3·01); p=0·006	1·24 (0·76–2·03); p=0·39	1·26 (0·75–2·10); p=0·38	1·0 (ref)
Parental separation (n=1095)§	2·28 (1·42–3·63); p≤0·001	1·88 (1·08–3·27); p=0·025	1·98 (1·36–2·89); p≤0·001	2·00 (1·30–3·05); p≤0·001	1·82 (1·32–2·51); p≤0·001	1·78 (1·27–2·48); p≤0·001	1·06 (0·77–1·46); p=0·71	1·10 (0·79–1·53); p=0·58	1·0 (ref)
Interpartner violence between parents (n=980)§	1·95 (1·14–3·35); p=0·016	1·70 (0·96–3·00); p=0·069	1·69 (1·09–2·61); p=0·019	1·59 (0·99–2·56); p=0·056	2·04 (1·46–2·85); p≤0·001	1·96 (1·39–2·76); p≤0·001	1·05 (0·73–1·52); p=0·79	1·03 (0·71–1·52); p=0·86	1·0 (ref)
Bullying (n=662)	1·92 (1·11–3·31); p=0·02	1·75 (0·98–3·12); p=0·059	1·57 (0·98–2·54); p=0·062	1·47 (0·90–2·43); p=0·13	1·13 (0·76–1·68); p=0·56	1·11 (0·73–1·68); p=0·62	0·75 (0·49–1·14); p=0·18	0·76 (0·51–1·15); p=0·20	1·0 (ref)
Physical abuse (n=714)§	2·47 (1·50–4·06); p≤0·001	2·27 (1·31–3·93); p=0·004	1·75 (1·10–2·78); p=0·018	1·69 (1·04–2·75); p=0·034	1·47 (1·00–2·16); p=0·051	1·49 (1·00–2·21); p=0·048	1·23 (0·87–1·76); p=0·24	1·25 (0·87–1·80); p=0·23	1·0 (ref)
Sexual abuse (n=162)	0·43 (0·04–4·81); p=0·49	0·57 (0·06–5·75); p=0·63	1·74 (0·76–3·98); p=0·19	2·09 (0·85–5·10); p=0·106	2·05 (1·13–3·73); p=0·018	2·02 (1·10–3·72); p=0·024	0·94 (0·42–2·12); p=0·88	1·23 (0·55–2·74); p=0·62	1·0 (ref)
Emotional abuse (n=1006)§	2·84 (1·78–4·52); p≤0·001	2·44 (1·49–3·99); p≤0·001	1·53 (0·99–2·36); p=0·06	1·42 (0·90–2·24); p=0·14	1·91 (1·36–2·67); p≤0·001	1·84 (1·30–2·61); p≤0·001	1·13 (0·82–1·57); p=0·45	1·13 (0·81–1·60); p=0·47	1·0 (ref)
Emotional neglect (n=318)	1·20 (0·51–2·83); p=0·68	1·22 (0·51–2·92); p=0·65	0·98 (0·45–2·14); p=0·95	0·93 (0·42–2·10); p=0·87	0·88 (0·49–1·58); p=0·67	0·85 (0·47–1·54); p=0·601	0·57 (0·30–1·10); p=0·09	0·61 (0·32–1·17); p=0·14	1·0 (ref)

Correction for alpha inflation needs to be applied (p=0·002), for assessing the results based on p values. ACE=adverse childhood experience. RRR=relative risk ratio.

* Adjusted for sex, participant socioeconomic position age 0–12 years, mother and partner's mental health before or during pregnancy, mother and partner's substance use during pregnancy, financial difficulties during pregnancy, and participant polygenic risk for cannabis initiation.

† Coded as dummy variables; comparison group is all other levels of ACE exposure, including no ACEs.

‡ Results of omnibus Wald test for pairwise comparisons, conducted on fully adjusted models: p≤0·05.

§ Results of omnibus Wald test for pairwise comparisons, conducted on fully adjusted models: p≤0·001.

¶ Results of omnibus Wald test for pairwise comparisons, conducted on fully adjusted models: p≤0·01.

Before adjustment, risk for early persisting regular use was particularly high in those who reported parental substance use or abuse (RRR 7·16 [95% CI 4·38–11·68]; p<0·001) relative to the low or no cannabis use class. After adjustment, risk for early persisting regular use (relative to the low or no cannabis use class) was almost four times as high in participants who reported parental substance use or abuse (RRR 3·90 [95% CI 2·10–7·24]; p<0·001), twice as high in those who reported parental mental health problems (2·02 [1·26–3·24]; p=0·003), physical abuse (2·27 [1·31–3·93]; p=0·004) or emotional abuse (2·44 [1·49–3·99]; p<0·001), and increased in those who reported parental separation (1·88 [1·08–3·27]; p=0·025). There was weaker evidence indicating increased risk in those who reported interpartner violence between parents (RRR 1·70 [95% CI 0·96–3·00]; p=0·069) or bullying (1·75 [0·98–3·12]; p=0·059).

After adjustment, risk for later onset regular use was twice as high in participants who reported parental separation (RRR 2·00 [95% CI 1·30–3·05]; p<0·001) and was increased in those who reported physical abuse (1·69 [1·04–2·75]; p=0·034), relative to the low or no cannabis use class. There was weaker evidence indicating increased risk of later onset regular use in those who reported parental substance use or abuse (RRR 1·82 [95% CI 0·97–3·40]; p=0·061) and interpartner violence between parents (1·59 [0·99–2·56]; p=0·056), relative to the low or no cannabis use class.

After adjustment, risks for early persisting occasional use were more than twice as high in those who reported parental substance use or abuse (RRR 2·38 [95% CI 1·51–3·73]; p<0·001) and sexual abuse (2·02 [1·10–3·72]; p=0·024) relative to the low or no cannabis use class. Risks were higher in those who reported a parent with a criminal conviction (RRR 1·90 [95% CI 1·20–3·01]; p=0·006), parental separation (1·78 [1·27–2·48]; p<0·001), interpartner violence between parents (1·96 [1·39–2·76]; p<0·001), and emotional abuse (1·84 [1·30–2·61]; p<0·001) relative to the low or no cannabis use class. There was weaker evidence that risks were increased in those who reported physical abuse (RRR 1·49 [95% CI 1·00–2·21]; p=0·048) relative to the low or no cannabis use class. Both before and after adjustment, only parental substance use or abuse was associated with risk of later onset occasional use (unadjusted RRR 2·02 [1·34–3·05], p<0·001; adjusted RRR 1·81 [95% CI 1·15–2·83], p=0·01) relative to the low or no cannabis use class.

To explore the effect of the covariates on the association between ACEs and cannabis use, all models were adjusted for separate covariates (appendix pp 24–32). The majority of attenuation of the effect of ACE exposure on cannabis use class occurred for the associations with early persisting regular use (unadjusted and adjusted results in table 3); adjusting for separate covariates identified that the majority of attenuation of this effect was due to controlling for the effects of maternal or partner substance use during pregnancy, and maternal or partner mental health before pregnancy. Although it is plausible there would be multicollinearity between the covariates of polygenic risk and parental substance use, adjustment for cannabis initiation polygenic risk score did little to attenuate the results, which indicates that any multicollinearity is unlikely to have led to over-adjustment (appendix pp 28–29).

**Figure 3:**
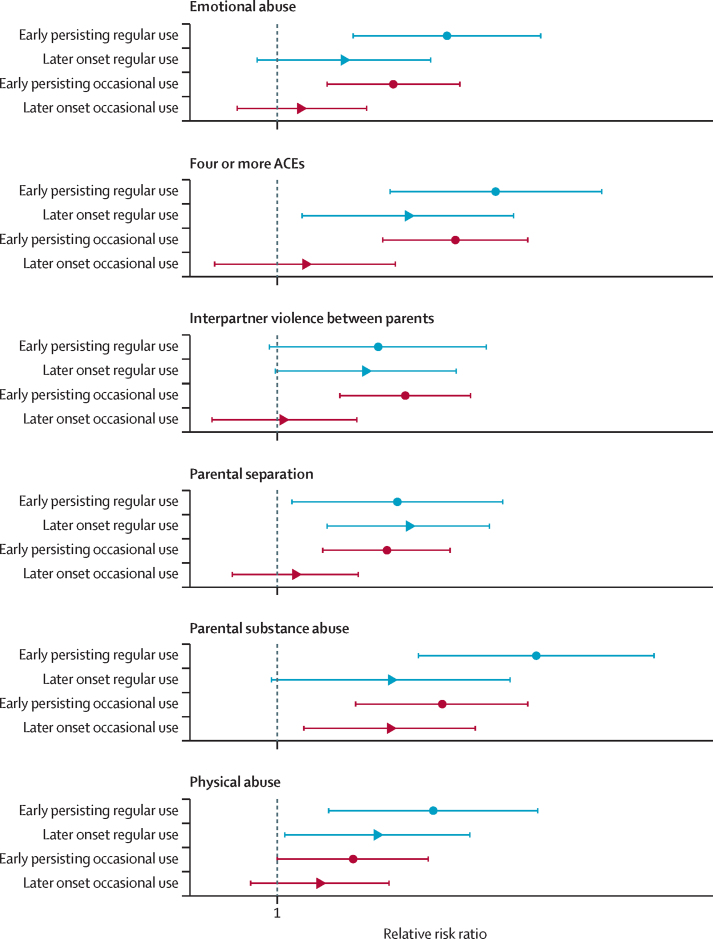
Effect sizes of the exposures for which omnibus pairwise comparisons indicated overall differences between cannabis trajectory classes Data are relative risk ratios and error bars are 95% CIs. ACE=adverse childhood experience.

Omnibus Wald tests of pairwise comparisons indicated significant differences between classes for the exposures of 4 or more ACEs, parental substance use or abuse, parental separation, interpartner violence between parents, physical abuse, and emotional abuse (table 3). The effect sizes for these classes are visualised in figure 3.

## Discussion

There was evidence of a dose–response association between ACEs and cannabis trajectories. Experiencing 4 or more ACEs was associated with more than three times the risk of early persisting regular use, more than twice the risk of early persisting occasional use, and twice the risk of later onset regular use. Parental substance use or abuse was associated with increased risk of cannabis use, with highest risks for early persisting regular use. Risks of early persisting regular use, plausibly the most problematic pattern of cannabis use, were also raised for those who had parental mental health problems, physical abuse, and parental separation. Physical abuse and parental separation were associated with an increased risk of all early onset and regular use classes.

We identified classes of cannabis use that plausibly represent risk for different levels of cannabis-related harms but we did not identify reduction in use by age 24 years. In relation to the previously published trajectories in the ALSPAC cohort using data only from ages 13–18 years,^27^ the present analyses identify an additional class of participants with later onset regular cannabis use. Older cohorts have identified groups in which cannabis use declines by the mid-20s.^12^, ^14^ This might reflect shifts in patterns of cannabis use among individuals born after the 1990s and delays in maturation out of persistent use.

Going through multiple ACEs between 0 years and 12 years increases the risks of early onset and regular cannabis use in adolescence. To our knowledge, no previous study has considered a composite measure of all 10 ACEs in relation to cannabis use. The current work adds to the evidence of a relationship between cumulative ACEs and negative physical and mental health outcomes.^6^

The long-term adverse effects of cannabis use might be greater when onset of use is early in adolescence, and when use is regular,^11^, ^12^ and several individual ACEs increased risks of early onset or regular cannabis use. Notably, parental substance use or abuse increased risks of early persisting regular and occasional cannabis use, and later regular cannabis use; this finding is consistent with previous studies that identified that parental history of drug use is associated with the likelihood of using cannabis at age 13–18 years.^17^ A substantial increase in the probability of early onset or regular cannabis use remained after adjusting for parent's substance use and mental health before birth, and for polygenic score for cannabis initiation. Previous studies focused on ACEs have not been able to control for genetic factors or for pre-birth parental mental health and substance use, which might impact on a child's mood and substance use or abuse regardless of whether the behaviours of the parents continue into childhood.^18^, ^20^ The present study strengthens the evidence base by indicating that effects of direct exposure to ACEs during childhood are over and above the influence of the genetic and environmental factors measured in the present study; plausible pathways between this distal ACE exposure and adolescent substance use or abuse include substance use availability, parenting practices, and the potential neurodevelopmental effects on offspring of exposure to substances (alcohol and tobacco, which were not controlled for).^20^ Such elements should be considered in future research into modifiable targets for intervention. The present work strengthens the evidence for early focus on parents (especially those engaged in substance use or abuse) to improve substance use outcomes in children.

Strengths of this study include considering both timing and frequency of cannabis use with repeated measures throughout adolescence, and repeated prospective and retrospective measures of ACEs maximises capture of adversity. The longitudinal nature of the data allows improved consideration of causality by ensuring the exposure precedes the outcome and confounder exposure precedes the exposure and outcome. However, there are several limitations. First, setting exposure to ages 0–12 years aimed to improve causal inference by ensuring the exposure preceded our outcome measure but might have missed proximal effects of exposure to ACEs occurring during adolescence. Second, ACEs might be under-reported due to their sensitive nature, which would lead to underestimation of effects. Third, attrition within ALSPAC means those who were included in this nested study were more likely to be White, female, and more affluent than the population from which the participants were originally drawn from.^23^ In addition to these limitations in the study sample, we were unable to provide non-aggregated data on Black and minority ethnicity categories. Fourth, the study attempted to account for the potential influence of genetic factors in the analyses. Although the cannabis initiation polygenic risk score did associate with cannabis use classes, the cannabis initiation polygenic risk score accounts for only a small amount of the variability in cannabis use.^25^ We have been unable to control for all potential confounding factors due to data availability and, therefore, cannot rule out that at least part of the observed association is due to unmeasured confounding. Fifth, we have had to assume that the ACEs data are missing at random for the purposes of imputation. By including data at multiple timepoints and auxiliary variables we have endeavoured to produce an accurate imputation model, and prevalence of ACEs did not differ largely between the imputed and the complete-case data, but we cannot rule out systematic bias in non-response to ACEs data. Sixth, it is notable that parental mental health problems were present in almost half of the participants in the sample. It has been previously acknowledged that this prevalence was higher than other ACE studies, but this finding is still in line with lifetime mental health prevalence estimates in the USA^28^ and Northern Ireland.^29^ Finally, although there were strengths to the detailed measures of ACEs available in ALSPAC, the present analysis was unable to account for timing and duration of ACEs. It is plausible to hypothesise that, for some ACEs, there might be an increased effect on substance use or abuse if exposure is proximate to the onset of substance use or abuse. Similarly, there might be differential effects of some exposures on substance use (eg, parental mental health problems, parental substance use or abuse) depending on the length of time for which they occur during childhood. Although we accounted for the co-occurrence of ACEs by using the ACE score, other clustering approaches, such as latent class analysis, could have been applied to the data. Nonetheless, previous work with this cohort has shown poor model fit of the latent class approach for ACEs,^30^ and this makes examining the association of latent classes of ACEs in relation to our latent classes of cannabis trajectories statistically and computationally challenging. Thus, we made the decision to use the ACEs score as our exposure, despite its known limitations.

The current work strengthens the evidence that experiencing multiple and specific ACEs is associated with increased risks for both regular and early cannabis use in adolescence. Previous work^6^ has shown that experiencing multiple ACEs raises risks for negative mental health outcomes; future work would benefit from considering ACEs as a confounder in the association between cannabis use and mental health outcomes. Alternatively, consideration should be given to whether engagement in regular cannabis use during adolescence might mediate the association between multiple ACEs in childhood and negative mental health outcomes, which would indicate that intervention on adolescent cannabis use might be a target to weaken the effects of ACEs on mental health.

Public health efforts to reduce ACEs could reduce regular cannabis use during adolescence. Additionally, the present findings relating to parental substance use or abuse suggest further research should explore targeting intervention on children growing up in such households, especially given that the effects of exposure to this ACE on adolescent cannabis use remained after accounting for substance use or abuse before the child's birth, and genetic risk for cannabis initiation. Another avenue for future research would be supportive interventions across the life course; parenting interventions for substance use or abuse are targeted later in childhood (typically school-based interventions as the beginning of secondary school, a period of education which starts at approximately 13 years of age), but the age of exposure in the present study was to ACEs at age 0–12 years, suggesting that interventions targeted earlier in childhood might be promising. Consequently, research into early interventions with parents during pregnancy and the postnatal stages to reduce childhood exposures to ACEs might be beneficial; focus on such an early stage could be a promising step for substance use intervention.

## Data sharing

The informed consent obtained from ALSPAC participants does not allow for the data to be made freely available through any third party maintained public repository. However, data used for this Article can be made available on request to the ALSPAC Executive. The ALSPAC data management plan describes in detail the policy regarding data sharing, which is through a system of managed open access. Full instructions for applying for data access can be found here: http://www.bristol.ac.uk/alspac/researchers/access/. The ALSPAC study website contains details of all the data that are available (http://www.bristol.ac.uk/alspac/researchers/our-data/).

## Declaration of interests

ML is employed by Drug Science, which receives an unrestricted educational grant from a consortium of medical cannabis companies to further its mission, that is the pursuit of an unbiased and scientific assessment of drugs regardless of their regulatory class. All other authors declare no competing interests.
