# Supplementary material for: Adverse childhood experiences and adolescent cannabis use trajectories: findings from a longitudinal UK birth cohort
Source: Lancet Public Health. 2023 May 25;8(6):e442–52. doi: 10.1016/S2468-2667(23)00095-6 (PMC10751739; doi:10.1016/S2468-2667(23)00095-6)
Supplement: Supplementary appendix [file mmc1.pdf]

THE LANCET
Public Health

Supplementary appendix

This appendix formed part of the original submission and has been peer reviewed. We post it as supplied by the authors.

Supplement to: Hines LA, Jones HJ, Hickman M, et al. Adverse childhood experiences and adolescent cannabis use trajectories: findings from a longitudinal UK birth cohort. *Lancet Public Health* 2023; **8:** e442–52.

Exploring the Effect of Adverse Childhood Experiences on Trajectories of Cannabis Use During Adolescence: Findings from a Longitudinal UK Birth Cohort

Appendices and Supplementary Tables

Contents

Page	Item
1	Appendix 1: Summary table of literature
5	Appendix 2: Additional methods details
16	Supplementary table 1: Items in the ALSPAC test battery and their cannabis frequency level categorisation
17	Supplementary Table 2: Matrix of Pearson correlations between exposures in imputed data (N=5212)
17	Supplementary Table 3: Number of adversities experienced by those reporting four or more adversities
18	Supplementary Table 4: Distribution of individual ACEs by ACE count category (%)
19	Supplementary Table 5: Sample characteristics in non-imputed data for the excluded and complete-case participants
21	Supplementary Table 6: Patterns of missing data and distributions in complete case and imputed data
22	Supplementary Table 7: Prevalence of cannabis use amongst those attending all 9 waves of data collection age 13-24
23	Supplementary table 8: Comparing the model fit of classes derived with different levels of available waves of data
24	Appendix 3: Regression models with separate covariate adjustment

APPENDIX 1: SUMMARY TABLE OF LITERATURE

The following papers were identified through systematic search. We searched abstracts of journal articles on Medline, Psycinfo and Embase from inception for August 9th 2022, with no restriction on language. Epidemiological studies were included if they 1) used one or multiple of the 10 classic ACEs as the exposure 2) included adolescent cannabis use as an outcome 3) used a general population human sample. The search identified 15 papers, of which 2 were excluded.

Search terms were (“cannabis” or “marijuana”) and adolescen* and ("ACEs" or "adverse childhood experiences")”. Epidemiological studies were included if they 1) used one or multiple of the 10 classic ACEs as the exposure 2) included adolescent cannabis use as an outcome 3) used a general population human sample.

The search identified 15 papers, of which 2 were excluded due to not meeting inclusion criteria.

Paper details		Methods						ACEs measures included in analysis												Covariate adjustment	
Reference	Year	Sample	Sample N	Longitudinal study?	Measure of frequency or severity of cannabis use?	Timing of cannabis use?	Prospective ACEs data?	Sexual abuse	Physical abuse	Emotional abuse	Emotional neglect	Parent substance abuse	Parent mental health or suicide	Violence between parents	Parent separation	Bullying	Parent criminal conviction	Individual ACE effects	Dose-response effects	Genetic	Lifetime parent substance use
(1)	2022	USA high school students	20125	No	No	No	No	Yes	Yes	Yes	Yes	Yes	No	Yes	Yes	No	Yes	No	Yes	No	No
(2)	2022	USA school students	4980	No	No	Yes	No	Yes	Yes	Yes	No	Yes	Yes	Yes	No	No	No	No	Yes	No	No
(3)	2022	USA middle and high school students	555	Yes	No	No	No	Yes	Yes	Yes	No	Yes	Yes	Yes	No	No	No	Yes	No	No	No
(4)	2022	24-32 year olds (Add Health sample)	8712	Yes	Yes	Yes	No	ACE items used not stated in paper	ACE items used not stated in paper	ACE items used not stated in paper	ACE items used not stated in paper	ACE items used not stated in paper	ACE items used not stated in paper	ACE items used not stated in paper	ACE items used not stated in paper	ACE items used not stated in paper	ACE items used not stated in paper	No	No	No	No

(5)	2021	12-18 year olds with criminal justice involvement	271	No	No	No	No	Yes	Yes	Yes	Yes	Yes	Yes	Yes	Yes	Yes	Yes	No	Yes	No	No
(6)	2021	Hispanic adolescents (USA)	1399	Yes	Yes	Yes	No	Yes	Yes	Yes	No	Yes	Yes	Yes	Yes	No	Yes	No	Yes	No	No
(7)	2020	Participants age 12-19 (Add Health)	9421	Yes	Yes	No	No	Yes	Yes	Yes	Yes	Yes	Yes	No	Yes	Yes	Yes	No	Yes	No	No
(8)	2019	USA adolescents	465	Yes	Yes	Yes	Yes	Yes	Yes	Yes	No	Yes	Yes	Yes	No	Yess	Yes	No	Yes	No	No
(9)	2019	Participants age 13-19 years, USA	1053	No	No	No	No	No	No	No	No	Yes	Yes	Yes	Yes	No	Yes	No	Yes	No	No
(10)	2018	5th-11th grade students, USA	79339	No	No	Yes	No	Yes	Yes	Yes	No	Yes	No	Yes	No	No	No	No	No	No	No
(11)	2018	Pupils in 8th, 9th and 11th grade, USA	1E+05	No	Yes	No	No	Yes	Yes	Yes	No	Yes	No	Yes	No	No	Yes	Yes	Yes	No	No
(12)	2018	Participants age 12-20	9422	Yes	Yes	Yes	No	Yes	Yes	No	Yes	No	No	No	No	No	No	Yes	No	No	No

(13)	2017	Participants aged 24-32 (Add Health)	11279	Yes	Yes	No	No	Yes	Yes	Yes	No	No	No	No	No	No	No	No	Yes	No	No

APPENDIX 2: ADDITIONAL METHODS DETAILS

Study population

Pregnant women in the former Avon Health Authority in south-west England who had an estimated date of delivery between 1 April 1991 and 31 December 1992 were invited to take part via media campaigns and outreach through antenatal and maternity services. (14) This resulted in a cohort of 14 541 pregnancies and 13,988 children alive at 1 year of age. When the oldest children were approximately 7 years of age, an attempt was made to bolster the initial sample with eligible cases who had failed to join the study originally. The total sample size for analyses using any data collected after the age of seven is therefore 15,454 pregnancies, resulting in 15,589 foetuses. Of these 14,901 were alive at 1 year of age.(15) From an eligible sample of 20, 248 pregnancies, 15, 247 pregnancies were enrolled over multiple stages of recruitment, resulting in 75% of eligible pregnancies being recruited. (14) In comparison to a national sample, ALSPAC enrolled participants at age 16 were found to have higher educational attainment, were more likely to be white and less likely to be eligible for free school meals. (14)

There were 68 data collection time points between birth and age 18, including 34 child-completed questionnaires, 9 'focus' clinical assessments and 25 questionnaires about the child completed by the mother or other main caregiver. Response rates have previously been published in full;(14) during adolescence, 48% of the eligible sample completed all 12 measures, and 75% completed at least one measure.

The ALSPAC study website contains details of all the data available through a fully searchable data dictionary (http://www.bristol.ac.uk/alspac/researchers/our-data/). Study data were collected and managed using REDCap electronic data capture tools hosted at University of Bristol (16,17).

Measures

Exposure

Adverse Childhood Experiences Participants in ALSPAC were asked to report on exposure to ACEs at multiple time points. The process of identifying the relevant items from this longitudinal study, in which ACEs data were collected from birth-23 years, has been previously outlined (18), but briefly:

Text scanning and visual searching of the ALSPAC data dictionary, which includes mother, child (with the child as the study participant) and mothers partner reports (59608 variables), was conducted in 2017 to identify the ACEs variables. Identified items were excluded if they did not conform to ACE definitions or were unsuitable for dichotomisation. Variables were recoded to a binary yes/no based on pre-set criteria.

The majority (89%) of measures included were assessed contemporaneously through childhood/adolescence, but retrospective self-report measures collected when participants were in their 20s (several forms of abuse and neglect, parents being violent towards each other) were included to complement the prospective data. For instance, the sexual abuse rates prospectively reported by parents were much lower than those retrospectively self-reported by the participants.

ACEs were defined as (18):

Sexual abuse - Ever sexually abused, forced to perform sexual acts or touch someone in a sexual way

Physical abuse -Adult in family was ever physically cruel towards or hurt the child

Emotional abuse -Parent was ever emotionally cruel towards the child or often said hurtful/insulting things to the child
Emotional neglect -Child always felt excluded, misunderstood or never important to family, parents never asked or never listened when child talked about their free time
Parent substance use/abuse -Parent was a daily cannabis or any hard drug user, or, had an alcohol problem

Parent mental health problems or suicide -Parent was ever diagnosed with schizophrenia or hospitalised for a psychiatric problem, or, during the first 18 years of the child's life, parent had an eating disorder (bulimia or anorexia), used medication for depression or anxiety, attempted suicide or scored above previously established cut-offs for depression (Edinburgh Postnatal Depression Scale (EPDS) >12 [13])
Violence between parents -parents were ever affected by physically cruel behaviour by partner, or, ever violent towards each other, including hitting, choking, strangling, beating, shoving.
Parental separation -Parents separated or divorced
Bullying -Child was a victim of bullying on a weekly basis

Parent convicted of a criminal offence -Parent was convicted of a crime

Questions were asked at multiple time points. For physical abuse, 49 variables were identified of which 32 were prospective and 9 child reported. For sexual abuse, 12 variables were identified of which 7 were prospective and 5 child reported. For emotional abuse, 46 variables were identified of which 33 were prospective and 5 child reported. For emotional neglect, 23 variables were identified of which 20 were prospective and 20 child reported. For bullying,19 variables were identified of which all were prospective and child reported. For household substance abuse, 70 variables were identified of which 70 were prospective and 1 child reported. For violence between parents, 48 variables were identified of which 44 were prospective and none child reported. For parent mental health problems or suicide, 82 variables were identified of which 78 were prospective and 2 child reported. For parent convicted of a criminal offence, 25 variables were identified of which 21 were prospective and none child reported. For parent separation, 48 variables were identified of which 39 were prospective and 3 child reported.

Because the ACE measures were derived from multiple questionnaires and clinics over a long time period (birth–23 years), no participants had data on all of the individual questionnaire items, necessitating the use of multivariate multiple imputation. Ideally, we would impute missing values of each questionnaire item, but the lack of complete cases in combination with the high number of variables (>500 separate questions relating to ACEs up to age 23) led to convergence errors. Therefore, we adopted a pragmatic approach to imputation, adapted from the scale-level imputation method proposed by Enders(19). We derived a dichotomous construct indicating presence or absence of each ACE. If a participant responded to 50% or more of the questions related to a given ACE, we used these data to create the dichotomous indicator. If the participant responded to less than 50% of the questions, we set the dichotomous indicator to missing. This avoided inducing biases which may occur if sample eligibility is easier for some participants than others (e.g. only needing to respond yes once to be a case, but needing to respond no at all time points to be a non-case).

Appendix 2 Table 1: Items included for ACE variables for ALSPAC data (reproduced with permission from 10.12688/wellcomeopenres.14716.1)

Adverse childhood experience (ACE)	Phrasing	Criterium dichotomisation	Retrospective
**Sexual abuse**	Sexually abused	yes	no
	When growing up someone molested respondent (sexually)	yes	yes (asked at 23yrs)

	Touched in a sexual way by adult or older child, or was forced to touch adult or older child in a sexual way, before age of 11	yes	yes (asked at 22yrs)
	Touched in a sexual way by adult or older child, or was forced to touch adult or older child in a sexual way, between ages of 11 and 17	yes	yes (asked at 22yrs)
	Adult or older child forced, or attempted to force, respondent into any sexual activity by threatening or holding respondent down or hurting respondent in some way, before age of 11	yes	yes (asked at 22yrs)
	Adult or older child forced, or attempted to force, respondent into any sexual activity by threatening or holding respondent down or hurting respondent in some way, between ages of 11 and 17	yes	yes (asked at 22yrs)
**Physical abuse**	Partner/respondent was physically cruel to child	yes	no
	Adult in family pushed, grabbed, shoved/smacked to discipline respondent, before age of 11	often	yes (asked at 22yrs)
	Adult in family pushed, grabbed, shoved/ smacked to discipline respondent, between ages 11 and 17	often	yes (asked at 22yrs)
	When growing up people in respondent's family hit them so hard that it left them with bruises or marks	yes	yes (asked at 23yrs)
	Adult in family kicked, punched, hit respondent (so hard it left bruises or marks), before age of 11	yes	yes (asked at 22yrs)
	Adult in family kicked, punched, hit respondent (so hard it left bruises or marks), between ages 11 and 17	yes	yes (asked at 22yrs)
**Emotional neglect**	Carer knows who friends are	never	no
	Carer asks/starts conversation about free time/ what happened at school	never	no
	Carer takes time to listen when teenager talks about what happened in free time	never	no
	Discuss problems with anyone in their family	very difficult	no

	Parent/carer talked about child's experiences at school/ friends/ things that are troubling	never	no
	Child feels left out of things	always	no
	Understood by parents	not	no
	When growing up there was someone to take respondent to the doctor if needed	never	yes (asked at 23yrs)
	Someone in family made child feel important or special, before 11	never	yes (asked at 22yrs)
	Someone in family made child feel important or special, between ages 11 and 17	never	yes (asked at 22yrs)
	Carer knows what child does with other children	nothing	no
**Emotional abuse**	Partner/respondent was emotionally cruel to child	yes	no
	Adult in family shouted/ said hurtful or insulting things to respondent, before age of 11	Very often	yes (asked at 22yrs)
	Adult in family shouted/ said hurtful or insulting things to respondent, between the ages of 11 and 17	Very often	yes (asked at 22yrs)
**Parental separation**	Parent reports divorce/separation	yes	no
	Your parents have divorced/separated	yes	no
	Parent still has the same partner/husband	no	no
**Mental health and suicide (attempts) by parents**	Parent has hurt themselves on purpose	yes	no
	Parent has attempted suicide	yes	yes, the reported age can be retrospective
	Taken medication for anxiety or depression	yes	no
	Edinburgh Postnatal Depression Scale (EPDS)	>12	no
	Schizophrenia	yes, either current or ever	Both
	Bulimia, anorexia nervosa	yes, recently	Both
	Ever admitted to hospital for psychiatric or mental health problems	yes	yes
**Parents violent towards each other**	Physically cruel	yes, affected them	yes, the reported age can be retrospective

	Aware of and affected by one 'parent' slapping, kicking, hitting or otherwise physically hurting the other	yes, ever	yes (asked at 21yrs)
	Kicked, bitten or hit each other	yes	no
	Physically twisted arm	yes	no
	Throw(n) bodily	yes	no
	Beaten each other up	yes	no
	Choke or strangle each other	yes	no
	threatened each other with knife	yes	no
	Used knife or other weapon on each other	yes	no
**Substance abuse in household**	Smoked cannabis	every day	no
	Hard drug use (including crack, heroin, amphetamine, opiate, cocaine, methadone, meth)	yes	no
	Hard drug addiction	yes, recently	no
	Alcoholism/ Drink problem	yes, ever / yes, saw doctor	no
	Alcohol Use Disorders Identification Test (AUDIT) score	>8	no
**Parent convicted**	Court conviction	yes	no
	Convicted of an offence	yes	yes, the reported age can be retrospective
**Bullying**	Overt bullying victim items including: personal belongings stolen, threatened/blackmailed, hit/beaten up	weekly	no
	Relational bullying victim items including: do something didn't want to, told lies about child	weekly	no
	Friends tried to get teenager to do things didn't want to / told lies about teenager	weekly	no
	Young person has been directly/relationally bullied	weekly	no
	Child has been bullied	all the time	no
	Upset by name calling/exclusion from groups or bullying	Most days	no

	Someone threatened/blackmailed teenager	weekly	no

Covariates

Maternal and/or Partner drug use during pregnancy (preceding ACE exposure). At 18 weeks gestation, mothers were asked if they had smoked cannabis or used any of amphetamine, barbiturates, crack, cocaine, heroin, methadone, ecstasy or any other drugs during pregnancy, and partners were asked if they had used any of the previously stated drugs in the past 3 month. Report of use of any of these drugs was classified respectively as maternal or partner illicit drug use during pregnancy.

Maternal/Partner mental health prior to/during pregnancy (preceding ACE exposure). At 12 weeks gestation, mothers and their partners reported on whether they'd ever experienced bulimia, drug addiction, alcoholism, schizophrenia, anorexia nervosa, severe depression, or other psychiatric problem. Mothers and partners who responded 'yes' to any of these items were classified as having experienced a mental health problem prior to or during pregnancy.

Childhood Socioeconomic Position (SEP) Childhood SEP was assessed through the highest occupation of the participant's mother or father, dichotomised as low (partly skilled or unskilled occupations) or higher (professional/managerial/technical/skilled manual or non-manual occupation). Data were collected through parental report when participants were 8 months, 21 months, 33 months, 4 years and 8 years of age.

Financial difficulties. At 32 weeks gestation (preceding participant ACE exposure) mothers rating difficulty above 0 for any of affording food, clothes, heating, rent/mortgage and other things considered essential for the child s were categorised as experiencing financial difficulty.

Cannabis initiation polygenic score: Genetic data. ALSPAC genetic data were acquired using the Illumina HumanHap550 quad genome-wide single nucleotide polymorphism (SNP) genotyping platform from 9912 participants. Following quality control assessment and imputation, genetic data was available for 7977 ALSPAC individuals. Polygenic scores were created using the PLINK (v1.9) (28,29) 'score' command. Polygenic scores were based on SNPs meeting a p-value threshold ≤ 0.5 in the cannabis initiation GWAS.

ALSPAC participant genetic data were acquired using the Illumina HumanHap550 quad genome-wide single nucleotide polymorphism (SNP) genotyping platform from 9912 participants. ALSPAC mother genetic data were acquired using the Illumina Human660W-quad genome-wide SNP genotyping platform from 10,015 participant mothers.

Individuals were excluded from further analysis on the basis of gender mismatches, minimal or excessive heterozygosity, disproportionate levels of individual missingness (>1%), evidence of cryptic relatedness (>10% of alleles identical by descent) and being of non-European ancestry (assessed by multidimensional scaling analysis including HapMap 2 individuals). SNPs with a minor allele frequency (MAF) of < 1%, Impute2 information quality metric of < 0.8, a call rate of < 95% or evidence for violations of Hardy-Weinberg equilibrium (p value $< 5 \times 10^{-7}$) were removed. Imputation of the ALSPAC genetic data was performed following haplotype estimation using ShapeIT (v2.r644) which utilises relatedness during phasing. Imputation was performed using Impute V2.2.2 against the 1000 genomes reference panel (Phase 1, Version 3; all polymorphic SNPs excluding singletons), using 2,186 reference haplotypes (including non-Europeans). Following quality control assessment and imputation, restricting to 1 young person per family, and excluding individuals who had withdrawn consent, genetic data were available for 477,482 SNPs for 7,977 ALSPAC participants and 8,251 ALSPAC participant mothers.

Polygenic Risk Scores (PRS) PRS for lifetime cannabis use were constructed for each ALSPAC individual using participants' genetic data and results of a lifetime cannabis use genome-wide association study (GWAS) meta-analysis (International Cannabis Consortium (ICC) and UK Biobank (n = 184 765)) (20) as a training set. Note that as ALSPAC formed part of the ICC GWAS sample, GWAS meta-analysis results were re-estimated after removal of the ALSPAC sample to avoid bias created by sample overlap. Prior to construction of scores, SNPs were removed from the analysis if they had a minor allele frequency less than 0.01, an imputation quality less than 0.8 or if there was allelic mismatch between ALSPAC and GWAS samples. Due to the high linkage disequilibrium (LD) within the extended major histocompatibility complex (MHC; chromosome 6: 25-34Mb) only a single SNP was included to represent this region. SNPs were pruned for LD using the PLINK 'clump' command to remove SNPs in LD ($r^2 > 0.25$) with a more significant SNP in the training set. Windows of 500kb were used to assess inter-SNP LD for pruning. PRS were then calculated using the PLINK (v1.9) (21,22) 'score' command. This command creates an average, weighted score for each individual based on the number of SNP effect alleles the individual has, multiplied by the magnitude of the GWAS association value for that SNP. Polygenic scores were constructed using SNPs meeting a p-value threshold ≤ 0.5 and weighted using the log(OR) for each SNP as reported in the lifetime cannabis use GWAS meta-analysis. (20)

For use as an auxiliary variable in the multiple imputation prior to analysis, lifetime cannabis use PRS were also created for ALSPAC participant mothers based on results of the ICC cannabis initiation GWAS (23) after removal of ALSPAC from the ICC GWAS meta-analysis.

Analysis

Missing data imputation. The rationale behind imputation of the ACE variables has been outlined previously (18). Briefly, individuals who had responded to <50% of ACE items were coded as missing for binary ACE measures (see above). Separate imputation models were performed for males and females. Each imputation model included all exposures, covariates and the outcome trajectory classes. The following variables were included in the present analysis as auxiliary variables for imputation of ACEs and covariates: maternal smoking during pregnancy, maternal homeownership status during pregnancy, maternal marital status during pregnancy, maternal weight prior to birth, age of mother at birth, maternal education, participant birthweight, weeks' gestation at delivery, parity, participant relationship separation at age 18, frequency of anti-depressant use at age 18, mother and partner's depression scores during pregnancy, participant depression score at age 18, participant AUDIT depression score at age 18, and father's AUDIT score (collected in participant's adulthood). Maternal PRS for cannabis was included as an auxiliary variable to facilitate imputation of participant cannabis initiation PRS.

Summary variables for experiencing any ACEs, or four or more ACEs, were derived following multiple imputation from the individual ACE measures. Estimates were obtained by pooling results across 40 imputed datasets using Rubin's rules (24).

References

1. Boccio CM, Meldrum RC, Jackson DB. Adverse childhood experiences and adolescent nicotine and marijuana vaping: Findings from a statewide sample of Florida youth. Prev Med. 2022 Jan 1;154:106866.

2. Clements-Nolle KD, Lensch T, Drake CS, Pearson JL. Adverse childhood experiences and past 30-day cannabis use among middle and high school students: The protective influence of families and schools. Addict Behav. 2022 Jul 1;130:107280.

3. Garduno LS. How Influential are Adverse Childhood Experiences (ACEs) on Youths?: Analyzing the Immediate and Lagged Effect of ACEs on Deviant Behaviors. J Child Adolesc Trauma. 2022 Sep 1;15(3):683–700.

4. Rajapaksha RMDS, Filbey F, Biswas S, Choudhary P. A Bayesian learning model to predict the risk for cannabis use disorder. Drug Alcohol Depend. 2022 Jul 1;236:109476.

5. Folk JB, Ramos LMC, Bath EP, Rosen B, Marshall BDL, Kemp K, et al. The Prospective Impact of Adverse Childhood Experiences on Justice-Involved Youth's Psychiatric Symptoms and Substance Use. J Consult Clin Psychol. 2021 Jun;89(6):483–98.

6. Rogers CJ, Forster M, Grigsby TJ, Albers L, Morales C, Unger JB. The impact of childhood trauma on substance use trajectories from adolescence to adulthood: Findings from a longitudinal Hispanic cohort study. Child Abuse Negl. 2021 Oct;120:105200.

7. Moss HB, Ge S, Trager E, Saavedra M, Yau M, Ijeaku I, et al. Risk for Substance Use Disorders in young adulthood: Associations with developmental experiences of homelessness, foster care, and adverse childhood experiences. Compr Psychiatry. 2020 Jul;100:152175.

8. Fava NM, Trucco EM, Martz ME, Cope LM, Jester JM, Zucker RA, et al. Childhood adversity, externalizing behavior, and substance use in adolescence: Mediating effects of anterior cingulate cortex activation during inhibitory errors. Dev Psychopathol. 2019 Oct;31(4):1439–50.

9. Stalgaitis CA, Wagner DE, Djakaria M, Jordan JW. Understanding Adversity and Peer Crowds to Prevent Youth Health Risks. Am J Health Behav. 2019 Jul 1;43(4):767–80.

10. Chatterjee D, McMorris B, Gower AL, Forster M, Borowsky IW, Eisenberg ME. Adverse Childhood Experiences and Early Initiation of Marijuana and Alcohol Use: The Potential Moderating Effects of Internal Assets. Subst Use Misuse. 2018;53(10):1624–32.

11. Duke NN. Adolescent Adversity and Concurrent Tobacco, Alcohol, and Marijuana Use. Am J Health Behav. 2018 Sep 1;42(5):85–99.

12. Oshri A, Kogan SM, Kwon JA, Wickrama K a. S, Vanderbroek L, Palmer AA, et al. Impulsivity as a mechanism linking child abuse and neglect with substance use in adolescence and adulthood. Dev Psychopathol. 2018 May;30(2):417–35.

13. LeTendre ML, Reed MB. The Effect of Adverse Childhood Experience on Clinical Diagnosis of a Substance Use Disorder: Results of a Nationally Representative Study. Subst Use Misuse. 2017;52(6):689–97.

14. Boyd A, Golding J, Macleod J, Lawlor DA, Fraser A, Henderson J, et al. Cohort Profile: The ‘Children of the 90s’—the index offspring of the Avon Longitudinal Study of Parents and Children. Int J Epidemiol. 2013 Feb;42(1):111–27.

15. Northstone K, Lewcock M, Groom A, Boyd A, Macleod J, Timpson N, et al. The Avon Longitudinal Study of Parents and Children (ALSPAC): an update on the enrolled sample of index children in 2019. Wellcome Open Res [Internet]. 2019 Mar 14 [cited 2019 Nov 6];4. Available from: https://www.ncbi.nlm.nih.gov/pmc/articles/PMC6464058/

16. Harris PA, Taylor R, Minor BL, Elliott V, Fernandez M, O’Neal L, et al. The REDCap consortium: Building an international community of software platform partners. J Biomed Inform. 2019 Jul 1;95:103208.

17. Harris PA, Taylor R, Thielke R, Payne J, Gonzalez N, Conde JG. Research electronic data capture (REDCap)—A metadata-driven methodology and workflow process for providing translational research informatics support. J Biomed Inform. 2009 Apr 1;42(2):377–81.

18. Houtepen LC, Heron J, Suderman MJ, Tilling K, Howe LD. Adverse childhood experiences in the children of the Avon Longitudinal Study of Parents and Children (ALSPAC). Wellcome Open Res. 2018 Aug 30;3:106.

19. Enders CK. Applied missing data analysis. New York, NY, US: Guilford Press; 2010. xv, 377 p. (Applied missing data analysis).

20. Pasman JA, Verweij KJH, Gerring Z, Stringer S, Sanchez-Roige S, Treur JL, et al. GWAS of lifetime cannabis use reveals new risk loci, genetic overlap with psychiatric traits, and a causal influence of schizophrenia. Nat Neurosci. 2018 Sep;21(9):1161–70.

21. Chang CC, Chow CC, Tellier LC, Vattikuti S, Purcell SM, Lee JJ. Second-generation PLINK: rising to the challenge of larger and richer datasets. GigaScience. 2015;4:7.

22. Purcell S, Neale B, Todd-Brown K, Thomas L, Ferreira MAR, Bender D, et al. PLINK: a tool set for whole-genome association and population-based linkage analyses. Am J Hum Genet. 2007 Sep;81(3):559–75.

23. Stringer S, Minică CC, Verweij KJH, Mbarek H, Bernard M, Derringer J, et al. Genome-wide association study of lifetime cannabis use based on a large meta-analytic sample of 32 330 subjects from the International Cannabis Consortium. Transl Psychiatry. 2016 Mar 29;6:e769.

24. Rubin DB, editor. Multiple Imputation for Nonresponse in Surveys [Internet]. Hoboken, NJ, USA: John Wiley & Sons, Inc.; 1987 [cited 2021 Aug 23]. (Wiley Series in Probability and Statistics). Available from: http://doi.wiley.com/10.1002/9780470316696

Supplementary table 1: Items in the ALSPAC test battery and their cannabis frequency level categorisation

Categorisation of cannabis use in analyses	Item in ALSPAC test battery (age and source)								
	**13years** *Computer in clinic*	**14years** *Postal questionnaire*	**15years** *Computer in clinic*	**16years** *Postal questionnaire*	**17years** *Computer in clinic*	**18years** *Postal questionnaire*	**20 years** *Postal questionnaire*	**22 years** *Postal questionnaire*	**24 years** *Computer in clinic*
Do not use	"Have you ever tried cannabis?" = "No"	"Have you ever tried cannabis?" = "No"	"Have you ever tried cannabis?" = "No"	"Have you ever tried cannabis?" = "No"	Ever tried cannabis? = "No"	"Have you ever tried cannabis?" = "No"	"Have you ever tried cannabis?" = "No"	"Have you ever tried cannabis?" = "No"	"Have you ever tried cannabis?" = "No"
Occasional	If "yes" to stem, "How many times in the last 6 months?" = "1-3 times" or ">4 times"	Or, if "yes" to stem, "I have only ever tried cannabis once or twice" or "I used to sometimes use or take cannabis but I never do now"<br>If "yes" to stem, "I sometimes use or take cannabis but less often than once a week"	Or, if "yes" to stem, "I have only ever tried cannabis once or twice" or "I used to sometimes use or take cannabis but I never do now"<br>If "yes" to stem, "I sometimes take cannabis but less often than once a week"	Or, if "yes" to stem, "I have only ever tried cannabis once or twice" or "I used to sometimes use or take cannabis but I never do now"<br>If "yes" to stem, "I sometimes use or take cannabis butless often than once a week"	If "yes" to stem, cannabis use in past 12 months = "Monthly or less" or "2-4 times per month"	If "yes" to stem, "In the last 12 months, how often have you used cannabis?" = "Once or twice" or "Less than monthly" or "Monthly (but less than weekly)"	Or, If "yes" to stem, "In the last 12 months, how often have you used cannabis?" = "Not in the last 12 months"<br>If "yes" to stem, "In the last 12 months, how often have you used cannabis?" = "Once or twice" or "Less than monthly" or "Monthly (but less than weekly)"	Or, If "yes" to stem, "In the last 12 months, how often have you used cannabis?" = "Not in the last 12 months"<br>If "yes" to stem, "In the last 12 months, how often have you used cannabis?" = "Once or twice" or "Less than monthly" or "Monthly (but less than weekly)"	Or, If "yes" to stem, "In the last 12 months, how often have you used cannabis?" = "Not in the last 12 months"<br>If "yes" to stem, "In the last 12 months, how often have you used cannabis?" = "Once or twice" or "Less than monthly" or "Monthly (but less than weekly)"
Regular	If "yes" to stem, "How many times in the last 6 months?" = "1 x per week"	If "yes" to stem, "I usually use or take cannabis between one and six times a week" or "I usually use or take cannabis more than six times a week, but I don't use it every day" or "I usually use or take cannabis every day"	If "yes" to stem, "I usually use or take cannabis between one and six times a week" or "I usually use or take cannabis more than six times a week, but I don't use it every day" or "I usually use or take cannabis every day"	If "yes" to stem, "I usually use or take cannabis between one and six times a week" or "I usually use or take cannabis more than six times a week, but I don't use it every day" or "I usually use or take cannabis every day"	If "yes" to stem, cannabis use in past 12 months = "2-3 times per week" or "4+ times per week"	If "yes" to stem, "In the last 12 months, how often have you used cannabis?" = "Weekly" or "Daily or almost daily"	If "yes" to stem, "In the last 12 months, how often have you used cannabis?" = "Weekly" or "Daily or almost daily"	If "yes" to stem, "In the last 12 months, how often have you used cannabis?" = "Weekly" or "Daily or almost daily"	If "yes" to stem, "In the last 12 months, how often have you used cannabis?" = "Weekly" or "Daily or almost daily"

Supplementary Table 2: Matrix of Pearson correlations between exposures in imputed data (N=5212)

Variables	(1)	(2)	(3)	(4)	(5)	(6)	(7)	(8)	(9)	(10)
(1) Physical abuse	1.000									
(2) Sexual abuse	0.065	1.000								
(3) Emotional abuse	0.269	0.074	1.000							
(4) Emotional neglect	0.028	0.042	0.014	1.000						
(5) Bullying	0.050	0.026	0.039	0.073	1.000					
(6 Violence between parents	0.140	0.038	0.187	0.053	0.044	1.000				
(7) Household substance abuse	0.059	0.022	0.098	0.027	0.036	0.102	1.000			
(8) Parent being convicted of a criminal offence	0.088	0.019	0.081	0.019	0.050	0.079	0.071	1.000		
(9) Parent separation	0.069	0.056	0.162	0.050	0.044	0.168	0.108	0.071	1.000	
(10) Parent mental health problems or suicide	0.121	0.041	0.195	0.039	0.053	0.153	0.089	0.055	0.156	1.000

Note: Stata code for correlations across imputed datasets obtained from https://www.stata.com/statalist/archive/2010-07/msg01382.html, accessed 10th Feb 2023. Correlations were the same to the second decimal place in the complete case and imputed datasets

Supplementary Table 3: Number of adversities experienced by those reporting four or more adversities

Number of ACEs	% within the four+ ACEs group
4	59.99
5	25.53
6	11.02
7	2.68
8	0.76
9	0.01

Supplementary Table 4: Distribution of individual ACEs by ACE count category (%)

	1 ACE	2/3 ACEs	4+ Aces
Parent substance abuse<br>N = 469	4.87	12.95	33.33
Parent mental health/suicide attempt<br>N=2168	40.01	66.23	84.17
Parent criminal conviction<br>N=370	4.33	9.76	26.56
Parental separation<br>N=1095	11.66	34.90	62.31
Parent inter-partner violence<br>N=980	9.01	29.44	65.47
Bullying<br>N=662	10.20	19.37	33.46
Physical abuse<br>N=714	6.42	19.88	52.58
Sexual abuse<br>N=162	1.92	3.70	12.66
Emotional abuse<br>N=1006	7.30	30.95	70.20
Emotional neglect<br>N=318	4.26	9.39	17.99

Supplementary Table 5: Sample characteristics in non-imputed data for the excluded and complete-case participants

	Participants excluded due to cannabis measures only available at 13-18 or 20-24 N = 3236[1]		Participants with complete data on cannabis use N=911[1]		Participants with complete data on cannabis use and on all study variables N=382[1]	
**Study variable**	**Frequency**	**%**	**Frequency**	**%**	**Frequency**	**%**
Parent substance abuse*	211	10.74	53	6.26	21	5.50
Parent mental health/suicide attempt*	901	44.76	332	38.56	137	35.86
Parent criminal conviction*	140	6.82	46	5.34	24	6.28
Parental separation*	471	25.31	126	15.05	39	10.21
IPV*	354	21.11	123	15.55	51	13.35
Bullying*	297	15.06	99	11.09	54	14.14
Physical Abuse*	117	6.92	108	12.30	45	11.78
Sexual Abuse*	26	1.07	33	3.69	14	3.66
Emotional abuse*	318	18.92	149	17.35	62	16.23
Emotional neglect*	66	3.92	43	4.80	24	6.28
Covariates						

Sex - female	1347	41.63	611	67.07	261	68.32
Lower SES age 0-12	163	10.64	23	5.03	14	3.66
Mother's drug use during pregnancy	25	0.77	2	0.22	1	0.26
Mother's partner drug use during pregnancy	139	4.30	29	3.18	8	2.09
Mother's mental health problems prior/during pregnancy	416	12.86	77	8.45	31	8.12
Mother's partner mental health problems prior/during pregnancy	201	6.21	60	6.59	27	7.07
Financial difficulties during pregnancy	1871	57.82	440	48.30	199	52.09
**Additional data**						
Ethnicity – non-white	141	4.85	29	3.24	12	3.14

*Complete if at least 50% of time points were not missing data

[1]Sample size for each reported frequency and proportion will vary due to missing data within the study variables

Supplementary Table 6: Patterns of missing data and distributions in complete case and imputed data

Role in analysis	Variable	Proportion missing data	Prevalence/mean in complete case data	Prevalence/mean in imputed dataset
Exposure	Physical abuse	42.09%	13.45%	13.69%
	Sexual abuse	25.34%	2.88%	3.07%
	Emotional abuse	43.52%	18.96%	19.36%
	Emotional neglect	41.79%	5.71%	6.07%
	Bullying	38.95%	12.34%	12.67%
	Household substance abuse	39.02%	8.35%	8.99%
	Violence between parents	46.89%	17.62%	18.76%
	Parent mental health problems or suicide	38.52%	40.61%	41.61%
	Parent separation	41.92%	19.70%	20.88%
	Parent being convicted of a criminal offence	36.66%	6.76%	7.08%
Covariate	Maternal drug use during pregnancy	0%	0.63%	-
	Partner drug use during pregnancy	0%	4.72%	-
	Maternal mental health prior to pregnancy	0%	10.69%	-
	Partner mental health prior to pregnancy	0%	5.93%	-
	Financial difficulties during pregnancy	0%	53.93%	-
	Childhood socioeconomic status	53.6%	7.48% (low SES)	7.31% (low SES)
	Cannabis initiation PRS	28.49%	.0022 (S.D 1.00)	.0005 (S.D 1.00)
	Gender	0%	39.91% (male)	-

Supplementary Table 7: Prevalence of cannabis use amongst those attending all 9 waves of data collection age 13-24 (N=911)

Cannabis use frequency	13 years	14 years	15 years	16 years	17 years	18 years	20 years	22 years	24 years
	N (%)	N (%)	N (%)	N (%)	N (%)	N (%)	N (%)	N (%)	N (%)
Do not use	894 (98.13)	901 (98.90)	870 (95.50)	844 (92.65)	793 (87.05)	785 (86.17)	675 (74.09)	703 (77.17)	709 (77.83)
Occasional	16 (1.76)	7 (0.77)	32 (3.51)	53 (5.82)	104 (11.42)	103 (11.31)	204 (22.39)	181 (19.87)	173 (18.99)
Regular	1 (0.11)	3 (0.33)	9 (0.99)	14 (1.54)	14 (1.54)	23 (2.52)	32 (3.51)	27 (2.96)	29 (3.18)

Supplementary table 8: Comparing the model fit of classes derived with different levels of available waves of data

Measures of model fit						
1+ waves of cannabis use frequency data available at 13-18 and 20-24 (N=5249)		3 classes	4 classes	5 classes	6 classes	7 classes
BIC		25328.89	25128.20	25025.92	25014.83	25055.15
Replicated loglikelihood		-12450.30	-12277.15	-12153.20	-12074.84	-12022.19
Entropy		0.77	0.78	0.79	0.76	0.77
LMR p-value		0.0001	0.0000	0.0004	0.0063	0.0048
BLRT p-value		0.0000	0.0000	0.0000	0.0000	0.0000
Smallest class size		308	241	135	139	55
2+ waves of cannabis use frequency data available at 13-18 and 20-24 (N=3642)		3 classes	4 classes	5 classes	6 classes	7 classes
BIC		18808.05	18666.99	18593.15	18589.70	18652.84
Replicated loglikelihood		-9200.28	-9060.48	-8954.29	-8883.29	-8845.59
Entropy		0.82	0.83	0.84	0.82	0.82
LMR p-value		0.0013	0.0054	0.0000	0.0038	0.2231
BLRT p-value		0.0000	0.0000	0.0000	0.0000	0.0000
Smallest class size		217	171	70	77	30
3+ waves of cannabis use frequency data available at 13-18 and 20-24 (N=1814)		3 classes	4 classes	5 classes	6 classes	
BIC		10534.98	10504.74	10493.238	10496.56	
Replicated loglikelihood		-5079.91	-5001.01	-4931.481	-4869.36	
Entropy		0.88	0.82	0.82	0.84	
LMR p-value		0.4229	0.0358	0.7118	0.9126	
BLRT p-value		0.0000	0.0000	0.0000	0.0000	
Smallest class size		63	45	46	42	

Appendix 3: Regression models with separate covariate adjustment

These tables are intended to supplement the fully adjusted models in the manuscript by presenting the analyses with adjustment for individual covariates. This allows the reader to observe the attenuating effects of the individual covariates.

Exposures and outcomes are as stated in the methods section of the main manuscript. Please note that composite dummy variables were derived of experiencing 0, 1, 2-3 or 4+ ACEs to explore dose-response effects of ACEs on cannabis use. These variables compare the stated number of ACEs reported against all other levels of ACE exposure, including reporting no ACEs.

The conceptualisation of the covariates is outlined in the methods section of the main manuscript.

Appendix table 1: Multinomial regression associations (RRR and 95% CI) between exposure to ACEs age 0-12 and cannabis use frequency latent class membership age 13-24, adjusted for mother/partner drug use during pregnancy

**Exposures age 0-12**	**Early persisting regular use N = 135**	**Later onset regular use N = 236**	**Early persisting occasional use N = 394**	**Later onset occasional use N=868**
	Adjusted RRR* (95% CI)	Adjusted RRR* (95% CI)	Adjusted RRR* (95% CI)	Adjusted RRR* (95% CI)
All other levels of ACE exposure, including no ACEs	1.0	1.0	1.0	1.0
One ACE[1] N=1508	0.62 (0.36 - 1.07)	0.64 (0.40 - 1.04)	0.76 (0.54 - 1.06)	0.95 (0.72 - 1.24)
Two-Three ACEs[1] N=1657	1.52 (0.96 - 2.41)	1.65 (1.12 - 2.44)	1.54 (1.15 – 2.08)	1.10 (0.84 - 1.45)
Four+ ACEs[1] N=542	3.64 (2.19 - 6.07)	2.11 (1.27 - 3.50)	2.49 (1.72 - 3.61)	1.15 (0.74 - 1.80)
Parent substance abuse N = 469	4.38 (2.48 - 7.75)	1.78 (0.94 - 3.37)	2.31 (1.48-3.60)	1.76 (1.16 - 2.69)
Parent mental health/suicide attempt N=2168	2.26 (1.45 - 3.52)	1.28 (0.89 - 1.83)	1.50 (1.13 - 1.98)	1.15 (0.90 - 1.47)

Exposures age 0-12	**Early persisting regular use N = 135**	**Later onset regular use N = 236**	**Early persisting occasional use N = 394**	**Later onset occasional use N=868**
	Adjusted RRR* (95% CI)	Adjusted RRR* (95% CI)	Adjusted RRR* (95% CI)	Adjusted RRR* (95% CI)
Parent criminal conviction N=370	1.28 (0.55 - 2.96)	1.24 (0.56 - 2.73)	1.90 (1.20 - 3.03)	1.26 (0.77 - 2.06)
Parental separation N=1095	1.94 (1.17 - 3.21)	1.94 (1.30 - 2.89)	1.75 (1.26 - 2.42)	1.04 (0.75 - 1.44)
Parent inter-partner violence N=980	1.78 (1.02 - 3.08)	1.58 (1.00 - 2.50)	1.98 (1.41 - 2.77)	1.03 (0.71 - 1.50)
Bullying N=662	1.93 (1.11 - 3.37)	1.56 (0.96 - 2.52)	1.13 (0.75 - 1.69)	0.75 (0.49- 1.14)
Physical abuse N=714	2.54 (1.52 - 4.24)	1.76 (1.10 - 2.82)	1.47 (1.00 - 2.17)	1.24 (0.87 - 1.77)
Sexual abuse N=162	0.47 (0.04 - 5.92)	1.68 (0.72 - 3.92)	2.12 (1.16 - 3.86)	0.98 (0.44 - 2.20)
Emotional abuse N=1006	2.63 (1.64 - 4.23)	1.48 (0.95 - 2.29)	1.85 (1.32 - 2.60)	1.13 (0.81 - 1.56)
Emotional neglect N=318	1.23 (0.53 - 2.87)	0.94 (0.43 - 2.02)	0.86 (0.48 - 1.56)	0.57 (0.30 - 1.10)

Appendix table 2: Multinomial regression associations (RRR and 95% CI) between exposure to ACEs age 0-12 and cannabis use frequency latent class membership age 13-24, adjusted for parent mental health

**Exposures age 0-12**	**Early persisting regular use N = 135**	**Later onset regular use N = 236**	**Early persisting occasional use N = 394**	**Later onset occasional use N=868**
	Adjusted RRR* (95% CI)	Adjusted RRR* (95% CI)	Adjusted RRR* (95% CI)	Adjusted RRR* (95% CI)
All other levels of ACE exposure, including no ACEs	1.0	1.0	1.0	1.0
One ACE[1] N=1508	0.61 (0.36 - 1.06)	0.63 (0.39 - 1.02)	0.75 (0.54 - 1.04)	0.93 (0.71 - 1.22)
Two-Three ACEs[1] N=1657	1.58 (1.00 - 2.49)	1.67 (1.13 - 2.46)	1.59 (1.19 - 2.13)	1.12 (0.86 - 1.47)
Four+ ACEs[1] N=542	3.63 (2.16 - 6.08)	2.18 (1.32 - 3.62)	2.64 (1.81 - 3.85)	1.15 (0.73 - 1.81)
Parent substance abuse N = 469	6.18 (3.69 - 10.34)	2.51 (1.47 - 4.27)	3.06 (2.01 - 4.66)	2.01 (1.32 - 3.04)
Parent mental health/suicide attempt N=2168	2.08 (1.32 - 3.27)	1.22 (0.84 - 1.77)	1.51 (1.13 - 2.01)	1.13 (0.88 - 1.46)
Parent criminal conviction N=370	1.23 (0.52 - 2.91)	1.28 (0.60 - 2.74)	1.90 (1.21 - 2.99)	1.24 (0.76 - 2.03)
Parental separation N=1095	2.10 (1.32 - 3.36)	1.94 (1.32 - 2.85)	1.80 (1.30 – 2.50)	1.06 (0.77 - 1.46)
Parent inter-partner violence N=980	1.82	1.66	2.04	1.04

Exposures age 0-12	Early persisting regular use N = 135	Later onset regular use N = 236	Early persisting occasional use N = 394	Later onset occasional use N=868
	Adjusted RRR* (95% CI)	Adjusted RRR* (95% CI)	Adjusted RRR* (95% CI)	Adjusted RRR* (95% CI)
	(1.06 - 3.14)	(1.06 - 2.58)	(1.46 - 2.85)	(0.72 - 1.50)
Bullying<br>N=662	1.81<br>(1.04 - 3.14)	1.5<br>(0.95 - 2.50)	1.12<br>(0.75 - 1.66)	0.75<br>(0.49 - 1.15)
Physical abuse<br>N=714	2.17<br>(1.29 - 3.65)	1.68<br>(1.06 - 2.68)	1.44<br>(0.98 - 2.12)	1.23<br>(0.86 - 1.76)
Sexual abuse<br>N=162	0.30<br>(0.02 - 6.09)	1.66<br>(0.73 - 3.79)	2.03<br>(1.10 - 3.73)	0.94<br>(0.41 - 2.13)
Emotional abuse<br>N=1006	2.49<br>(1.55 - 4.02)	1.45<br>(0.93 - 2.26)	1.88<br>(1.34 - 2.65)	1.12<br>(0.80 - 1.56)
Emotional neglect<br>N=318	1.21<br>(0.50 - 2.92)	0.97<br>(0.44 - 2.12)	0.87<br>(0.48 - 1.58)	0.57<br>(0.30 - 1.09)

Appendix table 3: Multinomial regression associations (RRR and 95% CI) between exposure to ACEs age 0-12 and cannabis use frequency latent class membership age 13-24, adjusted for cannabis PRS

**Exposures age 0-12**	**Early persisting regular use N = 135**	**Later onset regular use N = 236**	**Early persisting occasional use N = 394**	**Later onset occasional use N=868**
	Adjusted RRR* (95% CI)	Adjusted RRR* (95% CI)	Adjusted RRR* (95% CI)	Adjusted RRR* (95% CI)
All other levels of ACE exposure, including no ACEs	1.0	1.0	1.0	1.0
One ACE[1] N=1508	0.59 (0.35 - 1.02)	0.63 (0.39 - 1.01)	0.75 (0.53 - 1.04)	0.94 (0.72 - 1.24)
Two-Three ACEs[1] N=1657	1.68 (1.07 - 2.63)	1.70 (1.15 - 2.51)	1.58 (1.18 - 2.12)	1.13 (0.86 - 1.48)
Four+ ACEs[1] N=542	4.25 (2.60 - 6.94)	2.31 (1.42 - 3.74)	2.61 (1.81 - 3.78)	1.16 (0.74 - 1.81)
Parent substance abuse N = 469	7.19 (4.40 - 11.76)	2.58 (1.50 - 4.45)	3.07 (2.03 - 4.65)	2.02 (1.34 - 3.04)
Parent mental health/suicide attempt N=2168	2.37 (1.52 - 3.68)	1.29 (0.90 - 1.84)	1.51 (1.14 - 2.00)	1.15 (0.90 - 1.47)
Parent criminal conviction N=370	1.30 (0.56 - 3.00)	1.32 (0.63 - 2.76)	1.88 (1.20 - 2.95)	1.22 (0.75 - 2.00)
Parental separation N=1095	2.26 (1.41 - 3.61)	1.99 (1.36 - 2.90)	1.81 (1.30 - 2.51)	1.06 (0.77 - 1.46)

Exposures age 0-12	Early persisting regular use N = 135	Later onset regular use N = 236	Early persisting occasional use N = 394	Later onset occasional use N=868
	Adjusted RRR* (95% CI)	Adjusted RRR* (95% CI)	Adjusted RRR* (95% CI)	Adjusted RRR* (95% CI)
Parent inter-partner violence N=980	1.91 (1.11 - 3.29)	1.69 (1.09 - 2.62)	2.01 (1.44 - 2.82)	1.04 (0.72 - 1.50)
Bullying N=662	1.95 (1.12 - 3.37)	1.57 (0.97 - 2.55)	1.14 (0.77 - 1.71)	0.76 (0.49 - 1.15)
Physical abuse N=714	2.48 (1.51 - 4.08)	1.76 (1.11 - 2.79)	1.48 (1.00 - 2.17)	1.24 (0.87 - 1.77)
Sexual abuse N=162	0.43 (0.04 - 4.40)	1.68 (0.72 - 3.91)	1.95 (1.07 - 3.54)	0.91 (0.40 - 2.07)
Emotional abuse N=1006	2.80 (1.76 - 4.47)	1.53 (0.99 - 2.36)	1.89 (1.35 - 2.65)	1.12 (0.81 - 1.56)
Emotional neglect N=318	1.20 (0.51 - 2.85)	0.97 (0.45 - 2.12)	0.88 (0.49 - 1.58)	0.57 (0.29 - 1.10)

Appendix table 4: Multinomial regression associations (RRR and 95% CI) between exposure to ACEs age 0-12 and cannabis use frequency latent class membership age 13-24, adjusted for SEP

**Exposures age 0-12**	**Early persisting regular use N = 135**	**Later onset regular use N = 236**	**Early persisting occasional use N = 394**	**Later onset occasional use N=868**
	Adjusted RRR* (95% CI)	Adjusted RRR* (95% CI)	Adjusted RRR* (95% CI)	Adjusted RRR* (95% CI)
All other levels of ACE exposure, including no ACEs	1.0	1.0	1.0	1.0
One ACE[1] N=1508	0.59 (0.34 - 1.03)	0.63 (0.39 - 1.02)	0.74 (0.53 - 1.03)	0.92 (0.70 - 1.21)
Two-Three ACEs[1] N=1657	1.63 (1.04 - 2.55)	1.66 (1.12 - 2.44)	1.59 (1.18 - 2.14)	1.14 (0.87 - 1.50)
Four+ ACEs[1] N=542	4.06 (2.46 - 6.72)	2.18 (1.33 - 3.56)	2.65 (1.83 - 3.85)	1.23 (0.79 - 1.92)
Parent substance abuse N = 469	6.77 (4.11 - 11.17)	2.51 (1.48 - 4.28)	3.08 (2.03 - 4.67)	2.12 (1.40 - 3.21)
Parent mental health/suicide attempt N=2168	2.29 (1.47 - 3.57)	1.24 (0.87 - 1.78)	1.51 (1.13 - 2.01)	1.18 (0.92 - 1.51)
Parent criminal conviction N=370	1.29 (0.56 - 2.98)	1.28 (0.61 - 2.71)	1.91 (1.21 - 3.01)	1.26 (0.77 - 2.06)
Parental separation N=1095	2.13 (1.30 - 3.48)	1.90 (1.30 - 2.79)	1.83 (1.32 - 2.53)	1.08 (0.78 - 1.50)

Exposures age 0-12	Early persisting regular use N = 135	Later onset regular use N = 236	Early persisting occasional use N = 394	Later onset occasional use N=868
	Adjusted RRR* (95% CI)	Adjusted RRR* (95% CI)	Adjusted RRR* (95% CI)	Adjusted RRR* (95% CI)
Parent Inter-Partner violence N=980	1.86 (1.08 - 3.21)	1.63 (1.04 - 2.54)	2.04 (1.46 - 2.86)	1.07 (0.74 - 1.54)
Bullying N=662	1.90 (1.10 - 3.29)	1.55 (0.96 - 2.50)	1.11 (0.75 - 1.66)	0.76 (0.50 - 1.16)
Physical abuse N=714	2.40 (1.46 - 3.95)	1.72 (1.08 - 2.73)	1.47 (0.99 - 2.16)	1.24 (0.87 - 1.77)
Sexual abuse N=162	0.43 (0.05 - 3.87)	1.63 (0.72 - 3.72)	2.02 (1.10 - 3.69)	1.03 (0.47 - 2.28)
Emotional abuse N=1006	2.74 (1.72 - 4.38)	1.47 (0.94 - 2.29)	1.89 (1.35 - 2.66)	1.15 (0.83 - 1.60)
Emotional neglect N=318	1.12 (0.47 - 2.65)	0.95 (0.44 - 2.06)	0.88 (0.49 - 1.59)	0.57 (0.29 - 1.11)
